# Intrarenal resistive index conundrum: systemic atherosclerosis versus renal arteriolosclerosis

**DOI:** 10.1080/0886022X.2019.1674159

**Published:** 2019-10-10

**Authors:** Gabriel Ștefan, Cosmin Florescu, Alexandru-Anton Sabo, Simona Stancu, Gabriel Mircescu

**Affiliations:** a Dr. Carol Davila Teaching Hospital of Nephrology, University of Medicine and Pharmacy Carol Davila, Bucharest, Romania;; b Department of Anatomic and Molecular Pathology, Laboratoire National de Santé, Dudelange, Luxembourg;; c Nephrology Department, University of Medicine and Pharmacy Carol Davila, Bucharest, Romania

**Keywords:** Intrarenal resistive index, systemic atherosclerosis, renal arteriolosclerosis, kidney biopsy

## Abstract

**Background:** We aimed to evaluate the relationship between biopsy-proven kidney lesions, subclinical markers of atherosclerosis and intrarenal resistive index (RRI) in chronic kidney disease (CKD) patients.

**Methods:** This cross-sectional, single-center study prospectively enrolled 44 consecutive CKD patients (57% male gender, 54.1 (95%CI, 49.7–58.6) years, median eGFR 28.1 (15.0–47.7) mL/min) diagnosed by renal biopsy during 6 months in our clinic. RRI, carotid intima-media thickness (IMT), Kauppila score for abdominal aortic calcification (AACs) were assessed. Traditional and nontraditional atheroscleosis risk factors were also evaluated.

**Results:** Most of the patients had a diagnosis of glomerular nephropathy, with IgA nephropathy and diabetic nephropathy being the most frequent. RRI increased proportionally with CKD stages. Patients with RRI >0.7 (39%) were older, had diabetic and vascular nephropathies more frequently, higher mean arterial blood pressure, increased systemic atherosclerosis burden (IMT and AACs), higher percentage of global glomerulosclerois, GBM thickness, arteriolosclerosis and interstitial fibrosis/tubular atrophy. RRI directly correlated with age (rs = 0.55, *p* < 0.001) and with all the studied atherosclerosis markers (clinical atherosclerosis score rs = 0.50, *p* = 0.02; AACs rs = 0.50, *p* < 0.01; IMT rs = 0.34, *p* = 0.02). Also, global glomerulosclerosis (rs = 0.31, *p* = 0.03) and interstitial fibrosis/tubular atrophy (rs = 0.35, *p* = 0.01) were directly correlated with RRI. In multivariable adjusted binomial logistic regression models, only arteriolosclerosis was retained as independent predictor of RRI >0.7.

**Conclusion:** The analysis of RRI may be useful in the evaluation of the general vascular condition of the patient with CKD, supplying information about both microvascular and macrovascular impairment. Moreover, RRI correlates well with renal histopathologic characteristics, particularly with arteriolosclerosis.

## Introduction

The renal resistive index (RRI) measured by Doppler sonography in intrarenal arteries, describes the percentage reduction of end diastolic blood flow in renal vessels in relation to the maximal systolic blood flow. Data suggest that the RRI reflects the intrinsic state of vascular and parenchymal renal abnormalities. However, the resistive index also depends on central hemodynamic factors like the aortic pulse pressure and aortic stiffness [1–6]. The relative contributions of renal histologic features and central hemodynamic factors to the RRI remain unclear [7]. Moreover, the studies investigating the relationship between biopsy-proven kidney lesions and RRI had conflicting results. Some authors found that only arteriolosclerosis out of all histological parameters independently correlates with RRI, while others found that glomerulosclerosis and tubulointerstitial lesions are the strongest predictors of RRI [8–10].

Therefore, we performed a prospective study to evaluate in detail the relationship between biopsy-proven kidney lesions, subclinical markers of atherosclerosis and intrarenal resistive index in chronic kidney disease (CKD) patients.

## Methods

### Subjects

This prospective, cross-sectional study enrolled 44 consecutive patients referred to ‘‘Dr. Carol Davila’’ Teaching Hospital of Nephrology, Bucharest, Romania in a 6-month period, who underwent a diagnosis kidney biopsy.

Inclusion criteria were a biopsy-proven chronic kidney disease with a light microscopic specimen that included more than 5 glomeruli, age older than 18 years, and sufficient clinical data. Exclusion criteria were acute kidney injury, hepatic disease, valvular heart disease, renal artery stenosis, and urinary tract obstruction and refusal to give an informed consent.

The following data at kidney biopsy were available: age, mean arterial pressure (MAP defined as diastolic blood pressure plus 1/3 of pulse pressure), presence of hypertension (defined as a blood pressure >140/90 mmHg or the use of antihypertensive agents), inflammatory status (serum hemoglobin, erythrocyte sedimentation rate, C-reactive protein), lipid profile (serum cholesterol and triglycerides), serum albumin, eGFR (four variable CKD-EPI formula), proteinuria (24-h urine protein-to-creatinine ratio - UPCR) and hematuria (red blood cells per milliliter).

In order to evaluate the atherosclerotic burden, we used a semiquantitative score based on the clinical involvement of cerebral, carotid, coronary and peripheral vascular territories. Each affected territory was scored with one point; the maximum score was four. Patients were considered to suffer from coronary artery disease after coronary artery bypass surgery or in the presence of at least one diameter stenosis in coronary angiography; cerebrovascular disease if they had a previous ischemic stroke or a transient ischemic attack; carotid artery disease if they had previously undergone carotid surgery or if a significant stenosis could be detected by duplex sonography; peripheral artery disease was diagnosed after a previous peripheral revascularisation or if an ankle-brachial index of less than 0.9 could be detected in at least one pedal artery.

Abdominal aortic calcifications (AAC) were evaluated on a lateral lumbar X-ray (with the patient in standing position), as described by Kauppila [11].

For intima media thickness (IMT) determination B-mode ultrasonography imaging of the carotid artery was performed with a transducer frequency of 7 MHz (Samsung HM70A, linear transducer L7-16). Up to 4 cm of the common carotid artery, the carotid bifurcation and the internal carotid 2 cm distally from bifurcation were scanned bilaterally using longitudinal and transverse sections. IMT was defined as the distance between the leading edge of the first echogenic line (lumen-intima interface) and the second echogenic line (media-adventitia interface) in plaque-free arterial segments [12]. All measurements were performed by the same independent examiner under blind conditions.

For Doppler ultrasonography examination a real-time ultrasound device with color Doppler capacity (Samsung HM70A) and a 3.5 MHz convex-type transducer (CA1-7AD) were used. The examination was performed early in the morning, after 8-h overnight fast, with the patient in supine position and after at least 15 min rest. The signals were obtained from interlobar and arcuate arteries in the upper, middle, and lower parts of the kidney. The RRI was calculated as [(peak- systolic velocity – end-diastolic velocity)/peak systolic velocity]. The RRI value for each kidney was the mean of all 6 measurements. A mean RRI value was obtained for each patient by averaging the two kidneys’ mean RRIs (Spearman correlation coefficient between the two kidneys RRI measurements was 0,95, *p* < 0.001). The use of antihypertensive medication was not suspended before RRI measurement. Ultrasonographic examination that included RRI assessment was performed the day before the renal biopsy. In order to avoid inter-observer variability, all Doppler examinations were performed by the same examiner who was unaware of the study or the clinical details of the patients.

The study protocol was approved by the local Ethics Committee (registration number: 2017 LL UMF 07). All subjects signed an informed consent prior to any study procedure.

### Histological parameters

For each biopsy specimen, light microscopy, immunofluorescence and electron microscopy were routinely performed. One pathologist and one nephrologist reviewed independently the slides without knowledge of the original biopsy diagnosis, typically several months after the original pathology report.

The histological analysis included an in-depth review of the glomerular, tubulointerstitial and vascular compartments. Definitions of histologic variables used in our study were derived from the Mayo Clinic/Renal Pathology Society Consensus [13] and are depicted in Table 1.

**Table 1.** Histological variables.

	**Definition**
**Glomerular variable**	
Global glomerulosclerosis	Percentage of glomeruli with global glomerulosclerosis
Segmental glomerulosclerosis	Percentage of glomeruli with segmental glomerulosclerosis
Mesangial hypercellularity	Percentage of glomeruli with mesangial hypercellularity
Endocapillary hypercellularity	Percentage of glomeruli with endocapillary hypercellularity
Extracapillary hypercellularity	Percentage of glomeruli with extracapillary hypercellularity
Podocyte effacement	Presence of foot process effacement in EM (+/−)
Endothelial swelling	Presence of endothelial cell swelling in EM (+/−)
GBM thickness	Thickness of the GBM in EM (nm)
**Tubulo-interstitial variable**	
Interstitial fibrosis/tubular atrophy **Vascular variable ** Arteriolosclerosis Arteriosclerosis	Modfied Kliem classification: 0, absent; 0.5, focal area; 1, <10%; 2, 10–25%; 3, 25–50%; 4, >50%
	Presence of hyaline deposits in the wall of at least one preglomerular arteriole (+/−)
	absence of arteriosclerosis: normal intima thickness; moderate arteriosclerosis: thickening of intima with an intima/media ratio <1; severe arteriosclerosis: thickening of intima with an intima/media ratio ≥1.
Fibrinoid necrosis	Disruption of the GBM with fibrin exudation

EM: electron microscopy; GBM: glomerular basement membrane.

### Statistical analysis

Continuous variables are presented as mean or median and 95% confidence interval, according to their distribution, and categorical variables as percentages.

Patients were categorized into two groups based on the commonly used cutoff value of RRI of 0.7 [14]. Group comparisons were performed with Student’s t-test, χ^2^ test, and Mann–Whitney U test, as appropriate. The Pearson and Spearman test were used to assess correlations according to distribution.

The associations between RRI (defined as a dichotomous variable, higher or lower than 0.7) and IMT, AAC score, global glomerulosclerosis, interstitial fibrosis/tubular atrophy and arteriolosclerosis as covariates, adjusted for the traditional cardiovascular risk factors were investigated by multivariable adjusted binomial logistic regression.

A *p* < 0.05 was considered statistically significant.

Analyze-it (Analyze-it Software, Ltd., Leeds, UK) and SPSS (SPSS Inc., Chicago, IL, USA) software were used to analyze the data.

## Results

The mean age of the whole cohort was 54 years old, 57% were men. The median eGFR was 28 mL/min, and median proteinuria was 3.3 g/g creatinine. Approximately three quarters of the patients were treated for arterial hypertension and one quarter had diabetes mellitus (Table 2).

**Table 2.** Patient characteristics.

	All *N* = 44	RRI > 0.7 *n* = 17	RRI ≤ 0.7 *n* = 27	*p*
Age (years)	54.1 (49.7–58.6)	62.1 (57.4–66.7)	49.1 (43.0–55.3)	<0.001
Male gender (%)	57	65	52	0.4
Primary renal disease (%)				0.03
Glomerular nephropathy	75	53	89	
Diabetic nephropathy	14	24	7	
Vascular nephropathy**	7	18	0	
Tubulo-interstitial nephropathy	5	6	4	
Hypertension (%)	73	82	67	0.2
MAP (mmHg)	161.0 (151.7–170.4)	172.9 (156.2–189.7)	154.0 (143.0–165.0)	0.04
Diabetes mellitus (%)	20	35	11	0.05
Atherosclerosis score*	1 (0–1)	1 (1–2)	0 (0–1)	<0.001
Hemoglobin (g/dL)	11.50 (10.70–12.30)	10.54 (9.16–11.93)	12.07 (11.09–13.04)	0.06
Serum albumin (g/dL)	3.34 (3.11–3.57)	3.46 (3.11–3.81)	3.28 (2.96–3.59)	0.4
Cholesterol (mg/dL)*	218.0 (195.0–251.0)	200.0 (145.0–243.0)	237.0 (200.0–300.0)	0.07
Triglycerides (mg/dL)	195.9 (165.9–225.8)	174.0 (131.8–216.2)	208.8 (166.9–250.7)	0.2
Uric acid (mg/dL)	7.76 (6.98–8.54)	8.41 (6.84–9.97)	7.38 (6.49–8.26)	0.7
C reactive protein (mg/dL)*	6 (3–15)	9.5 (2–24)	4 (1–15)	0.3
ESR (mm/h)*	43.5 (22.0–77.0)	76.0 (22.0–97.0)	35.0 (17.0–72.0)	0.2
eGFR (mL/min)*	28.10 (15.06–47.76)	20.10 (13.60–47.76)	39.00 (15.05–72.80)	0.1
CKD class (%)				0.2
G1	12	0	19	
G2	14	13	15	
G3	23	19	26	
G4	21	31	15	
G5	30	38	26	
UPCR (g/g)*	3.32 (1.60–4.84)	2.69 (0.66–4.84)	3.68 (1.10–5.98)	0.4
UACR (g/g)*	1.99 (0.87–3.24)	1.94 (0.53–3.81)	2.11 (0.63–4.01)	0.6
Hematuria (mm^3^)*	45 (20–160)	70 (15–230)	30 (8–180)	0.4
Abdominal aortic calcification score	0 (0–3)	3 (0–6)	0 (0–1)	0.02
Intima media thickness (mm)	0.06 (0.06–0.07)	0.07 (0.06–0.08)	0.06 (0.06–0.08)	0.06
Renal morphometry				
Glomerulosclerosis global or focal (%)	68	82	59	0.1
Global glomerulosclerosis (%)*^†^	19.0 (12.7–25.4)	25.2 (14.3–36.1)	15.1 (7.1–23.2)	0.05
Focal glomerulosclerosis (%)*^†^	13.9 (6.7–21.0)	18.2 (4.8–31.7)	11.1 (2.5–19.8)	0.2
Endocapillary proliferation (%)	27	24	30	0.6
Extracapillary proliferation (%)	25	18	30	0.3
Arteriolosclerosis (%)	66	88	52	0.01
Severe arteriosclerosis (%)	14	24	7	0.1
Fibrinoid necrosis (%)	11	6	15	0.3
Interstitial fibrosis/Tubular atrophy*^‡^	1 (0.5–2)	2 (1–3)	1 (0.5–2)	0.03
GBM thickness (nm)*	420.0 (380.0–500.0)	475.0 (350.0–713.9)	400.0 (370.1–470.0)	0.05
Podocyte effacement (%)	71	56	80	0.1
Endothelial swelling (%)	59	75	48	0.08

*median.

**hypertensive nephropathy, atheroembolic renal disease.

^†^percentage of the total number of examined glomeruli.

^‡^Kliem classification modified: 0 absent; 0.5 focal area; 1 < 10%; 2 10–25%; 3 25–50%; 4 > 50%.

CKD: chronic kidney disease; eGFR: estimated glomerular filtration rate (CKD-EPI); ESR: erythrocyte sedimentation rate; GBM: glomerular basement membrane; MAP: mean arterial blood pressure; UAlb/Cr: urinary albumin to creatinine ratio; UP/Cr: urinary protein to creatinine ratio; RRI: renal resistive index.

Most of the patients had a diagnosis of glomerular nephropathy, with IgA nephropathy and diabetic nephropathy being the most frequent (Table 2).

**Figure 1.**
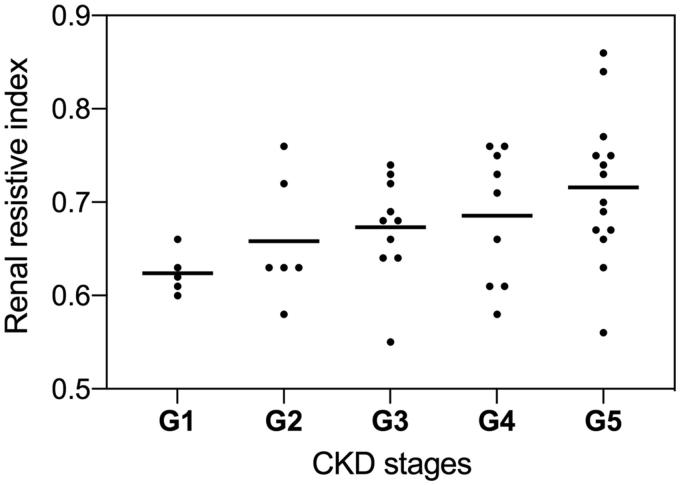
Renal resistive index (RRI) increased with chronic kidney disease (CKD) stages (G).

Intrarenal resistive index proportionally increased with CKD stages (Figure 1). Patients with abnormal RRI (39%) were older, had diabetic and vascular nephropathies more frequently, had higher mean arterial blood pressure, and increased systemic atherosclerosis burden (elevated atherosclerosis score, IMT and AAC score). Regarding renal morphometry, higher percentage of global glomerulosclerois, GBM thickness, arteriolosclerosis and interstitial fibrosis/tubular atrophy score were found in patients with RRI above 0.7 (Table 2).

**Figure 2.**
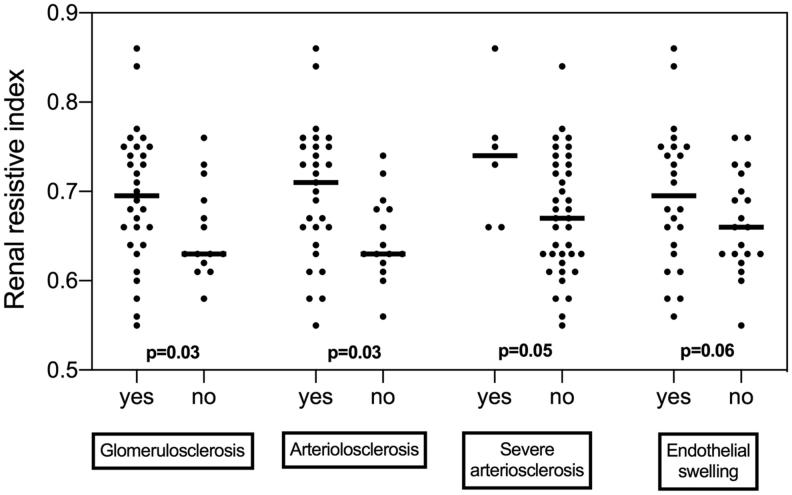
Patients with glomerulosclerosis (global and segmental), arteriolosclerosis, severe arteriosclerosis and endothelial swelling had higher renal resistive index.

Patients with global and segmental glomerulosclerosis, arteriolosclerosis, severe arteriosclerosis and endothelial swelling had higher RRI (Figure 2).

Intrarenal resistivity index was directly correlated with age and with all the studied atherosclerosis markers, atherosclerosis score, AAC score and IMT. Regarding the histopathologic parameters, global glomerulosclerosis and interstitial fibrosis/tubular atrophy were directly correlated with RRI. (Table 3).

**Table 3.** Correlation coefficients between RRI and investigated parameters.

	Correlation coefficient*	*p*
Clinical parameters		
Age (years)	0.55	<0.001
MAP (mmHg)	0.18	0.2
Kidney disease parameters		
eGFR (mL/min)	−0.34	0.02
UAlb/Cr (g/g)	−0.08	0.5
UP/Cr (g/g)	−0.14	0.3
Hematuria (mm^3^)	0.14	0.3
Systemic atherosclerosis parameters		
Atherosclerosis score	0.50	0.001
AAC score	0.50	<0.01
IMT (mm)	0.34	0.02
Histopathologic parameters		
Global glomerulosclerosis (%)^†^	0.31	0.03
Segmental glomerulosclerosis (%)^†^	0.16	0.3
GBM thickness (nm)	0.22	0.1
Interstitial fibrosis/Tubular atrophy^‡^	0.35	0.01
Arteriosclerosis^§^	0.18	0.2

*Pearson or Spearman according to distribution.

^†^percentage of the total number of examined glomeruli.

^‡^Kliem classification modified: 0 absent; 0.5 focal area; 1 < 10%; 2 10–25%; 3 25–50%; 4 > 50%.

^§^Arteriosclerosis score: 1, absent; 2, moderate; 3, severe.

AAC score: abdominal aortic calcification score; eGFR: estimated glomerular filtration rate (CKD-EPI); GBM: glomerular basement membrane; IMT: intima media thickness; MAP: mean arterial blood pressure; UAlb/Cr: urinary albumin to creatinine ratio; UP/Cr: urinary protein to creatinine ratio.

The association between RRI and atherosclerotic markers and categories of histopathologic lesions was tested separately in five distinct multivariable adjusted binomial logistic regression models, the remaining covariates - age, diabetes mellitus, arterial hypertension, eGFR, UACR - being the same. Only arteriolosclerosis was retained as independent predictor and made statistically significant contribution to the model (Table 4).

**Table 4.** Relationship between intrarenal resistivity index >0.7 and the studied atherosclerotic markers and histopathologic lesions in five separate models of multivariable adjusted binary logistic regression analysis.

Model	Variable	*β*	Exp(*β*) (95%CI)	*p*
AAC score*	Age	0.13	1.14 (1.03–1.25)	<0.001
	UAlb/Cr	−0.32	0.72 (0.50–1.02)	0.06
	Constant	−6.88	0.001	<0.01
IMT**	Age	0.08	1.09 (1.01–1.16)	0.01
	Diabetes mellitus	2.13	8.42 (1.19–59.47)	0.03
	eGFR	−0.02	0.97 (0.94–1.00)	0.05
	Constant	−4.76	0.009	0.02
Arteriolosclerosis^†^	Age	0.07	1.07 (1.00–1.15)	0.03
	Diabetes mellitus	2.58	13.19 (1.24–139.95)	0.03
	Arteriolosclerosis	2.42	11.28 (1.02–124.86)	0.04
	Constant	−7.07	0.001	<0.01
Global glomerulosclerosis^‡^	Age	0.08	1.09 (1.01–1.17)	0.01
	Diabetes mellitus	2.16	8.74 (1.23–61.74)	0.03
	eGFR	−0.03	0.97 (0.94–1.00)	0.05
	Constant	−4.88	0.008	0.01
Interstitial fibrosis/tubular atrophy^§^	Age	0.08	1.09 (1.01–1.17)	0.01
	Diabetes mellitus	2.16	8.74 (1.23–61.74)	0.03
	eGFR	−0.03	0.97 (0.94–1.00)	0.05
	Constant	−4.88	0.008	0.01

Variables entered at step 1 for each model: age, diabetes mellitus, arterial hypertension, eGFR, UAlb/Cr and AAC score or IMT or arteriolosclerosis or global glomerulosclerosis or interstitial fibrosis/tubular atrophy - depending on the model.

*Cox and Snell R^2^ = 0.37; Nagelkerke R^2^ = 0.50; (χ^2^=14.82; dif = 2; *p* = 0.001).

**Cox and Snell R^2^ = 0.34; Nagelkerke R^2^ = 0.46; (χ^2^ = 17.30; dif = 3; *p* = 0.001).

^†^Cox and Snell R^2^ = 0.36; Nagelkerke R^2^ = 0.49; (χ^2^ = 19.09; dif = 3; *p* < 0.001).

^‡^Cox and Snell R^2^ = 0.35; Nagelkerke R^2^ = 0.47; (χ^2^ = 18.11; dif = 3; *p* < 0.001).

^§^Cox and Snell R^2^ = 0.35; Nagelkerke R^2^ = 0.47; (χ^2^ = 18.11; dif = 3; *p* < 0.001).

## Discussion

We evaluated the relative contributions of renal lesions histopathologically assessed and subclinical atherosclerosis markers to the intrarenal resistivity index. To our knowledge this is the first report to address the full spectrum of intrarenal lesions. We found that RRI was associated with the atherosclerotic burden, global glomerulosclerosis, interstitial fibrosis/tubular atrophy and arteriolosclerosis. However, after adjustment for traditional cardiovascular risk factors only arteriolosclerosis was independently associated with RRI.

The studies on the relationship between histopathological findings and RRI in renal disease had conflicting results. Most of the studies did not include an evaluation of all renal compartments, i.e., glomeruli, tubulo-interstitium, and vascular. Moreover, histopathologic assessment was performed based only on a dichotomous scale or used vague criteria. In order to overcome these limitations, we studied all these components concurrently as damages are closely related to each other and their influence on RRI could be cumulative.

According to Ikee *et al*, only arteriolosclerosis out of all histologically studied parameters independently correlated with RRI in CKD [8]. Another study, performed in patients with renal parenchymal disease, showed that RRI was associated in order of significance with the degree of arteriolosclerosis, glomerulosclerosis, arteriosclerosis, edema and focal interstitial fibrosis [15]. Moreover, in 202 chronic kidney disease patients who underwent kidney biopsy, Hanamura *et al* found a significant relationship of RRI with glomerulosclerosis, arteriolosclerosis, and tubulo-interstitial damage [10].

Remarkably, arteriolosclerosis seems to be the only kidney biopsy finding that, in most of the studies, was independently correlated with the increase in RRI. Our results are in line with these observations: we found that only arteriolosclerosis was retained as an independent determinant of RRI after adjustment for traditional cardiovascular risk factors.

Hyaline arteriolosclerosis is a common vascular lesion, found in many different situations, including aging, arterial hypertension, diabetes mellitus, focal and segmental glomerulosclerosis. It is generated by the accumulation of serum proteins in the subendothelial space, often extending into the media [16]. Arteriolosclerosis appears to be related to the loss of glomerular autoregulation and, furthermore, to participate in the pathogenesis of the associated glomerular lesions [17].

In accord with other reports, we found that high RRI values are related to more severe tubulo-interstitial damage score, probably due to the increased stiffness of renal vessels due to interstitial fibrosis. Tubular atrophy and interstitial fibrosis correlate closely with renal function and long term prognosis, which may underlie the utility of RRI as an independent marker of renal and clinical outcome in CKD [9]. Moreover, in a recent large study on 992 patients, the tubulo-interstitial score was associated with intrarenal resistivity index [18]. However, despite the large cohort, the results could be hampered by the improper adjustment to covariates in multivariate analysis and by the histologic evaluation, limited only to glomerulosclerosis and tubulointerstitial damage scores [18].

In earlier reports, glomerulosclerosis association with increased RRI was not constant. Only one of the former studies reported a significant direct association of RRI with glomerulosclerosis. However, RRI was significantly lower when the glomerular involvement was isolated, i.e., not associated with any vascular and/or tubulo-interstitial damage [8,15,19,20]. In our cohort, global glomerulosclerosis and tubulo-interstitial lesions were directly correlated with RRI. Yet, global glomerulosclerosis might be an effect of senescence. Furthermore, in the multivariable analysis this associations were confounded by age, diabetes mellitus and renal function. Taken together, these findings suggest that RRI is not directly related to the glomerular lesions or to the extension of tubulo-interstitial damage.

As previously reported [15,19,21–23], we found a positive correlation between RRI and age. While in univariable analysis AAC score and IMT were related to RRI, after adjustment for the traditional cardiovascular risk factors this relationship disappeared. This suggests that the rise in RRI reflects more the vascular consequences of arterial hypertension and diabetes mellitus. This is in line with other reports, where RRI was considered a marker of target organ damage in essential arterial hypertension [24–26]. Furthermore, other studies demonstrated that high RRI was also associated with systemic atherosclerosis in diabetic patients [27,28].

In chronic kidney disease patients, many authors found a RRI of 0.70 or higher to be an independent risk factor for CKD progression regardless of the baseline eGFR, proteinuria, or arterial hypertension [29,30]. These results were confirmed in CKD patients at 2 and 4-years follow-up. Accordingly, we can assume that RRI of 0.70 or higher in chronic nephropathies could identify subjects with irreversible parenchymal damage [30,31]. In line with these results, we found that RRI increased with CKD stages. Moreover, proteinuria and hematuria were not related to RRI. Thus, arteriolosclerosis is likely to be the key culprit of a pathological RRI, since it is an irreversible lesion which impairs glomerular autoregulation [16].

Some limitations of this study should be acknowledged. First, the data resulted from a cross-sectional design with a small sample size from a single center, which can limit the statistical power. Second, the selection criteria for kidney biopsy influenced the composition of the cohort, i.e., we included mostly patients with glomerular nephropathies and only a few had vascular and tubulo-interstitial diseases. However, the final cohort reasonably describes the patients seen in daily practice. Finally, Doppler ultrasonography is an operator dependent method; nevertheless, the risk of inter-observer variability was reduced in our study because only one experienced examiner performed all ultrasound assessments.

In conclusion, the analysis of intrarenal resistivity index may be useful in the evaluation of the general vascular condition of the patient with CKD, supplying information about both microvascular and macrovascular impairment. Moreover, RRI correlates well with renal histopathologic characteristics, particularly with arteriolosclerosis.

## Ethics approval

All procedures performed in studies involving human participants were in accordance with the ethical standards of the institutional research committee at which the studies were conducted and with the 1964 Helsinki declaration and its later amendments or comparable ethical standards.

## Informed consent

Informed consent was obtained from all individual participants included in the study.

## Data Availability

The datasets used and/or analyzed during the current study are available from the corresponding author on reasonable request.
